# Determinants of Successful Public-Private Partnerships in the Context of Overweight Prevention in Dutch Youth

**DOI:** 10.5888/pcd10.120317

**Published:** 2013-07-11

**Authors:** Karlijn Leenaars, Monique Jacobs-van der Bruggen, Carry Renders

**Affiliations:** Author Affiliations: Karlijn Leenaars, Regional Public Health Service, GGD Hart voor Brabant, ’s-Hertogenbosch, the Netherlands; Monique Jacobs-van der Bruggen, Regional Public Health Service, GGD Hart voor Brabant, ’s‑Hertogenbosch, Academic Collaborative Centre for Public Health TRANZO, Tilburg University, Tilburg, the Netherlands; Carry Renders, Department of Health Sciences, Faculty of Earth and Life Sciences, EMGO Institute for Health and Care Research, VU University Amsterdam, Amsterdam, The Netherlands.

## Abstract

**Introduction:**

A public-private partnership (PPP) is an essential component of the Dutch community-based approach toward overweight prevention, Youth on Healthy Weight (JOGG). Beginning in 2010, 25 Dutch municipalities have implemented JOGG, but little is known about determinants of successful partnerships. This study aims to identify these determinants to guide other municipalities or communities in creating successful partnerships.

**Methods:**

Semistructured interviews were held in Veghel, a town in the southeast of the Netherlands, with private (n = 7) and public (n = 5) partners from the PPP involved in JOGG. We developed a themes and topics list that fit the purpose of our study. The interviews focused on the formation, functioning, and output of the partnership.

**Results:**

Recruitment of partners was facilitated by using preexisting networks. Corporate social responsibility, belief in the JOGG approach, importance of the health issue, and strengthened contacts with other partners were important motivations for partners to participate. In addition to partnership functioning and output, enthusiastic and decisive management, shared commitment, joint responsibility, and effective internal communication were important to the partners, as were clear goals and concrete actions to achieve these goals.

**Conclusion:**

To create successful partnerships, the program and its goals should appeal to the motivations of the partners. Involving partners in defining local program objectives can help to create shared commitment and joint responsibility. Further evaluation of partnerships’ impact on achieving program goals is a subsequent step to be taken to identify long-term determinants of successful PPPs.

## Introduction

In 2009, 13% of Dutch boys and 15% of Dutch girls aged 2 to 21 years were overweight, and 1.8% of boys and 2.2% of girls were obese (1). Childhood obesity is a complex problem that cannot be solved through any single strategy (2,3). An effective integrated approach of overweight prevention is likely to require action on many fronts, involve varied stakeholders, and be community-based (4–7). A successful approach is France’s Ensemble Prévenons l’Obésité Des Enfants (EPODE) (5), a large-scale, coordinated capacity-building approach for communities to implement effective and sustainable strategies to prevent childhood obesity. EPODE is based on 4 critical components: governmental support, public-private partnership (PPP), application of social marketing principles, and scientific guidance. The strength of EPODE lies in involving the whole society in combating childhood obesity. In the Netherlands, the EPODE approach was adopted and converted into Youth on Healthy Weight (JOGG) (8,9). Currently, 25 Dutch municipalities participate in JOGG (10).

A PPP is defined as a collaboration between private (for-profit) and public (nonprofit) sectors (11–13). A PPP can have a positive and innovative effect on achieving public health goals by leveraging the ideas, resources, and expertise of different partners (14,15). Private partners may bring new skills and funding to a partnership and enhance its scope of influence (16). Private parties have a growing interest in engaging in PPPs, because this engagement contributes to meeting corporate social responsibility (CSR) guidelines and the expectations of multiple stakeholders (12).

Within JOGG, private partners may contribute by contributing their expertise (eg, with respect to social marketing and communication), co-financing interventions, or organizing activities (10,11). However, the formation and functioning of PPPs within JOGG municipalities is new, and little is known about determinants for success. Therefore, this study aims to identify determinants for successful PPPs operating in the context of a community-based, integrated approach toward overweight prevention among Dutch youth. The results may guide other municipalities or communities in the creation of successful partnerships as part of such an integrated approach.

## Methods

Beginning in 2010, JOGG was first implemented in the Netherlands in 6 leading JOGG municipalities: The Hague, Rotterdam, Amsterdam, Utrecht, Zwolle, and Veghel. In comparison with the other municipalities, Veghel, a town with 26,513 inhabitants, has the most strongly developed PPP. Several major food companies are located in Veghel. The prevalence of overweight among youth in Veghel, about 10%, is lower than the national prevalence. In our prestudy, we conducted brief telephone interviews with project leaders of all leading Dutch JOGG municipalities (except Veghel) about the organization and functioning of the PPPs in their municipalities. In our main study, face-to-face interviews were held to evaluate the motivations, opinions, and experiences of private and public partners involved in the PPP of JOGG Veghel. We included participants who represented their organization in either the steering group, project group, or 1 of 4 working groups (communication, interventions, prevention and care, and research) of JOGG Veghel. We included 7 participants from private companies (5 owners or employees of 5 major food companies, 1 employee of an advertising agency, and 1 board member of an organization that represents 100 businesses in Veghel) and 5 participants from public organizations (2 employees of the municipality, a health educator from the Regional Public Health Service, a representative of primary schools, and an employee of a secondary school).

The interviews took place at the workplace of the participants and lasted approximately 1 to 1.5 hours. The interviews were performed by 1 of the researchers (K.L.). We used results from our prestudy and relevant literature (16–20) as a guide to develop a themes and topics list for the interviews (Table). The evaluation framework of Butterfoss (16), with its subsequent indicators, provided useful input for our list. This evaluation framework describes 3 levels for evaluating a PPP. Level 1 focuses on partnership infrastructure, function, and processes; level 2 evaluates partnership programs and interventions; and level 3 concerns health- and systems-level change outcomes. Indicators to measure and evaluate partnership formation, functioning, and output were considered useful. Audio-taped data were transcribed verbatim and manually coded by the first author (K.L.).

**Table:** Themes and Topics to Identify Determinants of Successful Public-Private Partnerships Operating in the Context of a Community-Based, Integrated Approach Toward Overweight Prevention in Dutch Youth

Themes	Topics	Topics Included in the Interviews
**Partnership’s formation**	Recruitment of partners	X
	Motivations to participate	X
	Organization of the public-private partnership	X

**Partnership’s functioning**	Collaboration	X
	Communication	X
	Management /Leadership	X

**Partnership’s output**	Partnership’s goals	X
	Action plans and interventions	X

**Partnership’s impact**	Perceived effectiveness	X
	Policy changes	X
	Changes in the physical/social environment	
	Changes in health indicators	

## Results

### Partnership formation

#### Recruitment of partners

JOGG Veghel resulted from a joint initiative of the municipality and 2 large companies in Veghel. Both the municipality and the companies regarded JOGG as a promising approach and a good way to generate positive national publicity for both the municipality and the companies. Subsequently, other companies were approached and asked to participate in JOGG. Initially, 4 major food companies were willing to participate. These companies and the municipality determined the scope of the program, its initial goals, and the contribution of the companies. The network of participating private partners enlarged when the Contact-group Veghel Companies (CVO), an organization representing the interests of approximately 100 medium-sized and large companies in Veghel, decided to participate. Through its cooperation with the CVO, Veghel can easily inform and approach many other companies to support JOGG Veghel whenever needed. However, this cooperation has not yet resulted in active involvement of new private partners in the partnership.

The private partners reported that the preexisting strong network in Veghel and good relationships between the companies were important contributing factors for engaging new private partners: “Maybe that’s a bit of the success of JOGG Veghel. Veghel has a business climate where companies have much contact with each other and the partners know each other well.” (Male participant, head of communications, age unknown). Because most of the public partners work in service of the municipality, and thanks to preexisting activities related to obesity prevention in Veghel in which public partners cooperated, involving these public partners in JOGG was easy.

#### Motivations of partners to participate in JOGG

Several reasons explain why the private and public partners were willing to participate in JOGG. First, participating in JOGG was considered as a suitable way to fill in the CSR policy of private partners, especially because most companies had health issues included in this CSR policy. In addition, the food companies felt a certain responsibility for the overweight problem in youth. Therefore, the opportunity to contribute to the program’s aim (overweight prevention) was regarded as a good reason to participate in JOGG. This rationale also applied to the public partners. Public partners, such as schools and the Regional Public Health Service “Hart voor Brabant,” have regular contact with the target group of JOGG, and they felt partially responsible for these young peoples’ health. They wanted to stimulate a healthy lifestyle as part of their caring role. Both public and private partners mentioned the proven effectiveness of the EPODE approach as a motivating factor, and these partners considered JOGG to be a promising approach. One potential private partner, however, had no faith in this approach and decided not to participate: “There must be a large, clear program where everyone says ‘Yes, this is it, and we are going to tackle it.’ But that is not there. And as long as it is not there, I think it is actually ridiculous to spend my best time at Veghel” (Male company owner, age 47).

Other motivations for the partners to engage in the partnership were strengthened contacts with other partners and the partners’ own interests. Private partners considered a good relationship with the municipality to be important, because Veghel is where the head offices of their companies are located. The self-interests of private partners were, for example, healthy employees (now and in the future) and strengthening of their companies’ image. For the public partners, strengthened contact with the municipality and the private partners was considered useful, and participation in the partnership facilitated their access to private partners. For schools, these contacts could be used to create internships for their students.

#### Organization of the PPP

The private and public partners contribute to JOGG in various ways. Some private partners contribute to JOGG financially (a structural amount per year) or through the supply of materials and goods. Companies agree not to use their financial contribution to JOGG for their own profit, and they cannot use participation in JOGG to promote their products. Private and public partners also contribute by providing manpower and knowledge. Representatives of the private and public partners participate in the steering, project, or working groups of JOGG. Public partners also are involved in the implementation of interventions. The municipality provides the project leader of JOGG, who coordinates the program. Different partners called the project leader “the linking pin”: “The municipality is a very important link in the process because it can bring the various partners together” (Female corporate affairs manager, age unknown).

### Partnership functioning

The collaboration of the public and private partners with the municipality was judged in various ways. The drive of the municipality to make the project successful and the enthusiasm and passion of the project leader were judged positive. Especially for the private partners, the shared responsibility between the municipality and the public and private partners was a reason to remain involved in the project. “You started this and that is a kind of shared commitment what you have. And the strength or success of JOGG lies precisely in that all these partners are together” (Male company owner, age 46) . Withdrawal of other partners was a potential reason for ending participation in the partnership. The public partners would appreciate more collaboration with the private partners in executing interventions.

Management and communication within the collaboration were most often mentioned as areas for improvement by the private and public partners. The private partners indicated that the management could be more decisive, and public partners preferred to have more guidance on the content area. An important comment of private partners was that “. . . we are not dependent on JOGG for achieving our own companies’ goals” (Male company owner, age 47) . The success of JOGG was not a primary concern for these partners, so having decisive management and good communication is essential to keeping these partners involved. “You don’t hear a thing. So the involvement gets lost, and I think that is a pity because of the energy and enthusiasm that the project leader has put into it” (Female communications advisor, age unknown). The various partners indicated that they heard little about the progress and were not informed about the status of JOGG. “I don’t know what happened, so if many things have happened, then one thing failed: communication about it” (Male company owner, age 46).

### Partnership output

#### Action plans and partnership’s goals

In Veghel, concept mapping was used to enhance the formulation of local goals. With this method, opinions of different people are mapped, stepwise, into a pictorial representation of the ideas of the group relative to the topic at hand (19). In Veghel, 15 stakeholders were involved through participation in a brainstorming session (to gather statements about individual JOGG objectives), execution of individual tasks (rating and prioritizing all objectives), and discussion and interpretation of results. The final concept map comprised 74 different statements about local JOGG objectives, showed how these objectives were related (statements were collapsed into 7 clusters), and showed the relative importance of each cluster. This method was considered useful. “The results will show what we consider to be important for Veghel, and all parties were allowed to contribute. The results will represent our joint objectives in which our individual objectives come together. I find that very interesting. From here we should work more effectively” (Female board member, age 44). 

The main goal of JOGG Veghel is to reduce the prevalence of overweight among youth from 9% in 2008 to 6% in 2015. Having a clear goal is important for the partners. “The nice thing is if we have an objective we get energy from it and then we go for it. And we really want to achieve the goal and we even think we can achieve more” (Female board member, age 44). Especially the private partners mentioned that their expectation of the partnership is to achieve the goal. The public partners are also satisfied if, for example, a good selection of interventions for the prevention of overweight would be created and structurally implemented in schools. Not achieving the program’s goal would be no reason for the partners to withdraw participation, because they have carefully considered participation and committed themselves to the project. In that case, changing the plan toward achieving the goal was a frequently mentioned, preferred solution.

#### Action plans and interventions

JOGG Veghel has been running for approximately 1 year, and several interventions and actions — mainly to promote healthful eating and physical activity — have occurred. Most of these interventions were organized and implemented by public partners. If private partners were involved, they were involved through sponsoring of goods and materials. Some partners were not satisfied with the partnership’s output so far: “There is no line, no structure, no vision, and no action plan” (Male company owner, age 47). Both private and public partners were clear about preferred actions. They wanted a concrete plan of action with clear interventions leading to the achievement of the program’s goals: “I think it's always nice that you really can make a good connection between the realized goal and the effort you put in to it” (Male company owner, age 46). A frequently heard comment of the private partners was that there is too much talking and not enough concrete action taking place. They want to work more effectively.

### Partnership’s effect

The partners did not think that JOGG has had a large effect on the community. “No, I don’t think so, but it’s a feeling I can’t prove. But it’s also a kind of program that requires more time” (Male head of communications, age unknown). The brand awareness of JOGG among young people was questioned. A few companies changed their policies as a result of JOGG. For example, some companies offer free fruit to their employees, or more healthful products are offered in company canteens. The partnership’s effect on changes in the physical or social environment or on health indicators was not assessed in this study.

## Discussion

This research aimed to identify determinants of successful PPPs as part of an integrated approach to combat overweight among Dutch youth. The results showed that recruitment of partners was facilitated by existing networks and cooperation between potential partners. CSR, belief in the JOGG approach, perceived importance of the health issue, a feeling of responsibility toward overweight prevention, and increased contacts with the other partners were important motivations to participate. Municipalities may fulfill an important role in the formation of partnerships because they can bring diverse partners together. Enthusiasm of the project leader, shared commitment, joint responsibility, and decisive management were important determinants for successful functioning of the partnership, as were effective internal communication, clear goals, and concrete plans and actions toward achieving these goals. Several other studies have found consistent results (12,20–23).

In Veghel, feelings of shared commitment and joint responsibility among partners were strengthened by involving partners in defining local goals. Concept mapping was useful to involve stakeholders in determining these goals (24). The resulting concept map also proved suitable for guiding the further implementation and evaluation of the local JOGG approach.

Our study has limitations. The results are based on self-reported, subjective views of a limited number of people. These people are still working together, which might restrain them from critical comments. Although our main study was limited to the evaluation of one PPP, results from our prestudy in 5 other municipalities were consistent with our final findings. The PPP of Veghel was considered to be a suitable case for the main study because the involvement of private partners in Veghel is relatively strong. Veghel agreed to cooperate and all major private and public partners participated in our study. At the time of the evaluation, the PPP of JOGG Veghel was functioning for approximately 1 year, and the organization structure, secondary goals, action plans, working processes, and people and partners involved were still being developed. Therefore, our evaluation focused on determinants that were relevant for the early stages of a partnership (ie, formation and functioning) but did not address indicators aimed to measure long-term partnership’s achievements (eg, changes in health indicators). Further research is needed to evaluate the functioning of PPPs in the longer term to identify determinants for successful partnerships with respect to their contribution to achieving long-term public health goals.

Prevention of obesity and lifestyle-related noncommunicable diseases (NCDs) is a challenge for public health. It is increasingly acknowledged that, because of the multisectoral nature of the risks for NCDs and obesity, cross-sector collaborative action is required for prevention and health promotion (25,26). Despite different perspectives and interests of public and private parties, public health issues have the ability to bring these parties together. Multisector partnerships are more likely to succeed in initiating essential policy and environmental changes and are increasingly used in the field of public health (25,27). Although our study focused on a PPP for overweight prevention, determinants for success such as clear goals and joint responsibility may also apply to other public-health related partnerships. Further research would be useful to see whether our results can be duplicated and whether those results would be more generalized or would need to be supplemented.

Our study showed that PPP formation can be facilitated by using preexisting networks, and the program and its goals should appeal to the motivations of potential partners. Related to partnership functioning and output, enthusiastic and decisive management, shared commitment, joint responsibility, effective internal communication, clear goals, and concrete actions were determinants of a successful PPP as part of an integrated approach toward overweight prevention among Dutch youth. The results of our study are useful for municipalities and communities who intend to form a new PPP as part of an integrated approach to address lifestyle problems. We recommend that they make use of local networks and appeal to the motivations of potential partners. Current JOGG municipalities may use the results to optimize their partnership’s functioning. For Veghel, in particular, managing more decisively, improving internal communication, and elaborating plans into concrete actions are highly recommended.
